# The Relationship between Dietary Polyphenol Intakes and Urinary Polyphenol Concentrations in Adults Prescribed a High Vegetable and Fruit Diet

**DOI:** 10.3390/nu12113431

**Published:** 2020-11-09

**Authors:** Erin D. Clarke, Megan E. Rollo, Clare E. Collins, Lisa Wood, Robin Callister, Mark Philo, Paul A. Kroon, Rebecca L. Haslam

**Affiliations:** 1 School of Health Sciences, Faculty of Health and Medicine, The University of Newcastle, Callaghan, NSW 2308, Australia; erin.clarke@uon.edu.au (E.D.C.); megan.rollo@newcastle.edu.au (M.E.R.); clare.collins@newcastle.edu.au (C.E.C.); 2 Priority Research Centre for Physical Activity and Nutrition, The University of Newcastle, Callaghan, NSW 2308, Australia; robin.callister@newcastle.edu.au; 3 Centre for Asthma and Respiratory Disease, Hunter Medical Research Institute, Rankin Park, NSW 2287, Australia; lisa.wood@newcastle.edu.au; 4 School of Biomedical Sciences and Pharmacy, Faculty of Health and Medicine, The University of Newcastle, Callaghan, NSW 2308, Australia; 5 Quadram Institute Bioscience, Norwich Research Park, Norwich NR4 7UQ, UK; mark.philo@quadram.ac.uk (M.P.); paul.kroon@quadram.ac.uk (P.A.K.)

**Keywords:** biomarker, urine, polyphenols, fruit, vegetables

## Abstract

Urinary polyphenol metabolites are potential biomarkers of dietary polyphenol intake. The current study aims to evaluate associations between total diet, vegetable and fruit polyphenol intakes with urinary polyphenol metabolite concentrations in a sample of adults prescribed a diet rich in vegetables and fruit. Thirty-four participants completed a 10-week pre-post study. Participants were asked to consume Australian recommended daily vegetable and fruit serves and attend measurement sessions at baseline and at weeks 2 and 10. Two 24-h diet recalls were collected at each time-point and polyphenol intakes were calculated using the Phenol-Explorer database. Spot urine samples, collected at each time-point, were analyzed for 15 polyphenol metabolites using liquid chromatography-mass spectroscopy. Spearman’s correlation analyzes assessed the strength of relationships between urinary and dietary polyphenols. Linear mixed models were used to investigate relationships between polyphenol excretion and intake. Total urinary polyphenols were significantly correlated with total polyphenol intakes at week 10 (*r*_s_ = 0.47) and fruit polyphenols at week 2 (*r*_s_ = 0.38). Hippuric acid was significantly correlated with vegetable polyphenols at baseline (*r*_s_ = 0.39). Relationships were identified between individual polyphenol metabolites and vegetable and fruit polyphenols. Linear mixed model analyzes identified that for every 1 mg increase in polyphenol intakes, urinary polyphenol excretion increased by 16.3 nmol/g creatinine. Although the majority of relationships were not sufficiently strong or consistent at different time-points, promising relationships were observed between total urinary polyphenols and total polyphenol intakes, and hippuric acid and vegetable polyphenols.

## 1. Introduction

Current dietary intake assessment methods rely on individuals either recalling previous intake using food frequency questionnaires (FFQs) or 24-h dietary recalls, or recording intake prospectively using weighed or measured food records [1,2,3]. Dietary intake is reported over varying periods of time for the purpose of quantifying food and/or nutrient intakes. All methods have associated systematic and random errors that contribute to over or under estimation of intake and may result in some inaccuracy when used to quantify the diet-disease relationships and/or effect sizes within dietary interventions [4,5]. Identification of appropriate objective biomarkers of dietary intake, synchronized with self-reported dietary intake, allows estimation of error associated with self-report methods. Once relationships between dietary intake and the biomarker of interest are established, further statistical modeling can be applied to calibrate results and estimate measurement error associated with dietary intake [6]. 

Polyphenols are phytonutrients found abundantly in plant-based foods, such as vegetables, fruit, cocoa, tea, coffee, alcohol and wholegrains [7]. Plant foods contain different types of polyphenols, including flavonoids and phenolic acids which comprise the most abundant types, but also minor components such as stilbenes, chalcones and others; polyphenols is the collective term for all these compounds [7]. Polyphenol metabolites have been identified in urine, suggesting that urinary polyphenols could be useful biomarkers to assess intakes of total polyphenols and polyphenol-rich food sources. Urinary polyphenol metabolites have been evaluated in research as a concentration biomarker for individual and group level polyphenol intakes, as well as used to reflect intakes of specific foods, food groups, vegetables, fruits and wholegrains [8,9,10,11,12,13].

Polyphenols are primarily absorbed in the large intestine where they are broken down via gut bacteria into smaller molecules and absorbed into the portal vein, then are transported attached to albumin to peripheral tissues or excreted via the kidneys in the urine [14]. The half-life of urinary polyphenol metabolites is 1–24 h and each metabolite may differ in the time to maximum concentration [15]. For example, quercetin has a half-life of approximately 10–28 h but a time to maximum concentration of 2–4 h [15,16]. Large variations can occur between the amount of polyphenol ingested and urinary concentrations, with 1% to 25% of total polyphenols consumed excreted in urine [17]. Many factors contribute to disparities identified between ingested and excreted polyphenols. For example, ingested polyphenols may not have been absorbed across the gut barrier, instead they may have been metabolized by gut bacteria in the large intestine or may have been taken up by peripheral tissues [17]. Bioavailability and absorption of polyphenols is influenced by numerous factors including age, sex, BMI, physical activity level, smoking status, the food matrix, seasonality, ripeness, food processing, cooking techniques, storage, the polyphenol’s chemical structure and the presence of specific gut microbiota [7,18,19,20]. While not all of these confounders can be accounted for, researchers need to consider them where possible through inclusion in statistical models. 

Urinary polyphenols, measured as concentrations of individual metabolites and total polyphenol metabolites have been used to assess intakes of vegetables, fruits, wholegrains, soy, coffee and tea, as well as total polyphenols [12,13]. Correlations between intakes of polyphenol rich foods and urinary polyphenol metabolites have varied, ranging from weakly to strongly positive [12,13]. While urinary polyphenols have been used to assess the intakes of polyphenol-rich foods, few studies have explored the application of spot urinary polyphenols in intervention studies, instead using 24-h urine samples. The application of spot urine samples is promising based on previous findings [21,22]. Spot urine samples are less burdensome on participants and easier to administer within study protocols, therefore further validation of their application is warranted. 

High intakes of vegetables and fruits delivers a diet rich in polyphenols, which has been associated with beneficial health effects, such as lower risk of inflammatory conditions including obesity, type 2 diabetes and cardiovascular disease [19,23]. The identification of a biomarker that reflects intakes of polyphenols and polyphenol-rich foods, particularly vegetables and fruits, would allow for a further investigation of relationships between polyphenol intakes and disease risks. However, further research is required to understand which urinary polyphenols or combinations of polyphenol metabolites can be used as objective measures of polyphenol intakes. This current study uses a combination of 15 individual urinary metabolites identified previously as potential biomarkers of vegetable, fruit and total polyphenol intakes [12].

Therefore, the aim of the current study was to evaluate associations between total polyphenol intakes and polyphenols derived from key vegetable and fruit sub-groups and urinary concentrations of polyphenol metabolites in a sample of Australian adults consuming a diet rich in vegetables and fruit. 

## 2. Materials and Methods 

### 2.1. Study Design

Participants were enrolled in a 10-week pre-post high vegetable and fruit weight loss intervention. Data were collected at baseline (pre), week 2 (early) and week 10 (post) intervention. Participants were advised to fast for approximately 12-h prior to data collection sessions. There was some non-compliance with fasting at week 2 however all results were still included. Measurements, including height and weight, a spot urine sample collected after the first morning void, demographic characteristics and dietary intake were collected at each session. 

Ethics approval was obtained from The University of Newcastle, Human Research Ethics Committee and written, informed consent obtained from all participants (H-2013-0315). Trial was registered with the Australian New Zealand Clinical Trials Registry (ACTRN12620000046909). 

### 2.2. Participants and Recruitment 

Between August 2017 and March 2018, participants were recruited through flyers and media releases distributed via The University of Newcastle and Hunter Medical Research Institute, NSW, Australia. Males and females aged 18–45 years with a BMI between 25–35 kg/m^2^ and body fat percentage of > 20% for males and > 30% for females that were willing to consume a high vegetable and fruit diet and interested in reducing body weight were included. A sample size of 35 participants was required to determine a statistically significant change in weight loss, which was the primary aim of the study. This sample size was calculated using a mean weight loss of 2.59 kg (SD 3.83), (unpublished data from the intervention from Williams et al. [24]), powered at 80% using a 5% significance level and a 30% drop out rate. Forty-three participants consented to participate, of whom 34 (79%) completed the study protocol.

### 2.3. Anthropometry 

At baseline, height was measured using a stadiometer (Holtain Harpenden, Crymych, UK) and recorded to 0.1 cm. Weight was measured (InBody 720; Biospace Co., Ltd., Seoul, South Korea) and reported to the nearest 100 g. Two measures were collected at each time point and averaged.

### 2.4. Dietary Assessment 

Dietary intake was assessed using repeat 24-h recalls collected using the Australian version of the Automated Self-Administered 24-h recall system (ASA-24) [25]. The 24-h recalls were completed approximately three days prior to and during each assessment session (i.e., two recalls at baseline, Weeks 2 and 10), totaling six 24-h recalls across the 10-week intervention. ASA-24 is an online dietary recall platform that uses eight passes to ensure that all foods and drinks are reported [25]. Information was collected on the individual foods recorded and gram weights or milliliters of food. Intake is reported as the average from both recalls. The ASA24-Australia uses the AUSNUT 2011–13 food and nutrient database for analysis [26]. 

Polyphenol intakes were calculated for each recall using the Phenol-Explorer database version 3.6 [27]. Foods were matched between items in the recalls and the Phenol-Explorer database with the most appropriate option chosen. Food matching was assessed based on food matching guidelines by the Food and Agriculture Organization [28]. Ingredients lists and recipes were deconstructed for mixed and packaged food items. If foods could not be matched it was assumed no polyphenols were present in that food item. Foods were classed as vegetables or fruits, then further classified into sub-groups, according to their AUSNUT food code [26]. Vegetable sub-groups were legumes and pulses (e.g., chickpeas, black beans, baked beans), peas and beans, carrot and similar root vegetables (e.g., sweet potato, beetroot, ginger), leaf and stalk vegetables (e.g., lettuce, spinach, celery, asparagus), brassica vegetables (e.g., cabbage, broccoli, cauliflower, bok choy), ‘other’ fruiting vegetables (e.g., capsicum, pumpkin, cucumber, zucchini) and ‘other’ vegetables (e.g., onion, garlic). Fruit sub-groups included pome fruits (e.g., apples, pears), citrus fruits (e.g., oranges, lemons, limes), berries (e.g., strawberries, raspberries, blueberries), tropical fruits (e.g., bananas, pineapple, mangoes) and ‘other’ fruits (e.g., grapes, kiwi fruit). 

### 2.5. Intervention 

Participants received dietary counselling from an Accredited Practicing Dietitian (EDC) at baseline and week 2. In these sessions, participants received individualized feedback on their current food and nutrient intakes based on a personalized report generated from the Australian Eating Survey, a 120-item FFQ [29]. Education on the health benefits of a high vegetable and fruit diet and tips on how to increase their vegetable and fruit intakes were provided. Participants were supported to set a goal related to increasing vegetable and fruit intakes and were encouraged to set two other nutrition-related goals, e.g., to decrease take away foods to once per week. At baseline, participants were also provided with a box containing 49 serves of vegetables (35 serves) and fruits (14 serves), equivalent to a one week supply based on national recommendations of 5 serves of vegetables and 2 serves of fruit daily, in order to visually demonstrate the target weekly amount. The vegetable and fruit box contained a mix of fresh, frozen and canned options (Supplementary Table S1). To compensate any additional costs of changing their diet, participants that completed the intervention also received a $100 gift card. Further details on the intervention have been reported elsewhere [30]. 

### 2.6. Urine Collection 

Spot urine samples were collected from participants at baseline, week 2 and week 10. Samples were collected in the session after the initial morning void and collected midstream into a sterile container and stored on ice until they could be frozen at −80 ℃. 

### 2.7. Urine Analysis

Enzyme hydrolysis was performed on principle urine samples to convert most of the glucuronidated and sulphated polyphenols to the aglycone forms. The extracted portion was then submitted for liquid chromatography-mass spectroscopy (LC-MS) analysis (Waters Acquity UPLC coupled to a TSQ-µ triple quadrupole mass spectrometer operated in electrospray mode) using targeted multiple reaction monitoring (MRM) detection for native compounds and their associated sulphate and glucuronide conjugates. 

Samples were prepared as described in Hollands et al. [31]. Commercially available standards were used for phloretin, naringenin, gallic acid, eriodictyol, epicatechin, quercetin, isorhamnetin, caffeic acid, ferulic acid, hippuric acid, creatinine and p-coumaric acid (Sigma-Aldrich, Poole, UK). Additional in-house synthesized standards of quercetin 3-O-glucuronide, quercetin 3-O-sulfate, 5-(3,4-dihydroxyphenyl)-y-valerolactone, 5-(3,4-dihydroxyphenyl)-y-valerolactone 3-sulfate and 5-(3,4-dihydroxyphenyl)-y-valerolactone 3-O-glucuronide were also used. No standards were available for epicatechin glucuronide and sulfate, therefore MRM tracking was achieved by calculating the addition of mass of a sulphate or glucuronide group to the native compound. It was assumed that MRM response factors for these compounds were comparable to the natives and thus quantified tentatively. For available standards, suitable MRM channels were optimized and responses against the calibration standards were used. A range of mixed standards was added to 200 µL of phosphate buffer for urine samples over a range of 10 to 2000 ng/mL. 

Frozen urine was warmed to room temperature and mixed briefly by vortex mixer before sampling. A subsample (200 µL) was taken and phosphate buffer (200 µL), sulfatase (80 µL, 80 U) and glucuronidase (80 µL, 800 U) added. The sample was vortexed and incubated for 2 h at 37 °C. Following incubation, taxifolin internal standard was added (10 µL of 10 µg/mL in methanol), along with dimethyl formamide (570 µL) and formic acid (40 µL). The sampled was vortex mixed and filtered (0.2 µm) prior to LC-MS analysis. A subsample of the final extract (10 µL) was diluted with 700 µL of phosphate buffer and taxifolin added (10 µL of 10 µg/mL in methanol). These samples were analyzed using LC-MS for hippuric acid and creatinine on a similar but shorter separation gradient. 

Polyphenol metabolites were chosen based on findings from a previous review [12] and additional input from M.P. and P.A.K. who conducted the analysis of the samples. Total urinary polyphenols refers to the total of a targeted subset and were calculated as the sum of: 5-(3,4-dihydroxyphenyl)-y-valerolactone (34DHVL), 5-(3,4-dihydroxyphenyl)-y-valerolactone 4-O-glucuronide (34DHVL-4-GlcA), 5-(3,4-dihydroxyphenyl)-y-valerolactone 3-O-glucuronide (34DHVL-3-GlcA), 5-(3,4-dihydroxyphenyl)-y-valerolactone 4-sulphate, 5-(3,4-dihydroxyphenyl)-y-valerolactone 3-sulphate (34DHVL-3S), quercetin, quercetin 3-O-glucuronide, quercetin-3-O-sulphate (Q3S), isorhamnetin, epicatechin, epicatechin glucuronide, epicatechin sulphate, phloretin, naringenin, gallic acid, eriodictyol, ferulic acid, caffeic acid and p-coumaric acid. Total urinary polyphenols were reported as polyphenols per gram of creatinine to standardize for differences in hydration between samples. 

Hippuric acid was also measured in urine samples. Hippuric acid excretion was reported separately to the other urinary polyphenols because as well as reflecting intakes of polyphenol-rich foods, it is a metabolite formed from non-polyphenol endogenous compounds, including aromatic amino acids such as phenylalanine, and was detected in amounts approximately 1000 times higher than the other measured polyphenols [32]. 

Metabolites not detectable in urine samples were 5-(3,4-dihydroxyphenyl)-y-valerolactone 4-sulphate, quercetin, quercetin 3-O-glucuronide, isorhamnetin and epicatechin glucuronide. The limit of quantification for these metabolites were 10 ng/mL, except for quercetin and isorhamnetin, which had a limit of quantification of 20 ng/mL. 

### 2.8. Statistical Analysis 

Data were firstly checked for normality to determine data distribution. All data were skewed and therefore are reported as median (interquartile range), unless stated differently. Only the participants who completed the study were included in the analysis. Intakes of polyphenols were reported using descriptive statistics and polyphenol intakes were reported by vegetables and fruits and their sub-groups to describe the main sources. Wilcoxon sign rank tests were used to assess whether polyphenol intakes and urinary polyphenols were significantly different from baseline to week 2 and week 10. 

Associations between self-reported intakes and each polyphenol biomarker were analyzed separately at baseline, 2 and 10 weeks by firstly assessing scatterplots and Spearman correlation coefficients. Correlations < 0.2 were classified as weak, 0.2–0.6 moderate and > 0.6 strong based on previous dietary biomarker studies [33,34,35].

Linear mixed models were used to investigate relationships between polyphenol intakes and urinary polyphenol and hippuric acid concentrations accounting for within and between individual differences. The models were run unadjusted and adjusted for confounders of age, energy intake, sex and BMI. Time was included in both models to account for the different time points (baseline, weeks 2 and 10 sessions) and any differences in energy intake and BMI. 

Analyzes were classified as statistically significant if *p* values were < 0.05 and all statistical analysis were undertaken using Stata version 14.2 (StataCorp, College Station, TX, USA). A sensitivity analysis to account for the multiple hypothesis testing of correlations between urinary polyphenols and polyphenol intakes was undertaken using the Bonferroni adjustment. The adjusted *p* value was determined by dividing the usual significance threshold (0.05) by the number of tests (18), therefore the new significance level was < 0.0028. 

## 3. Results

### 3.1. Dietary Polyphenol Intakes

Of the 34 completing participants, 53% were female and the median (IQR) age was 35 (15) years. Additional participant characteristics are summarized in Table 1. At baseline, median vegetable intakes were 3.97 (4.3) serves/day and fruit intakes were 0.98 (1.1) serves/day. Across the intervention vegetable intakes had a non-significant increase from baseline to week 2 (+0.37 (5.8) serves/day, *p* = 0.30) and week 10 (+0.73 (5.8) serves/day, *p* = 0.41). Fruit intakes increased significantly from baseline to week 2 (+0.77 (1.6) serves/day, *p* = 0.004) and week 10 (+0.50 (1.7) serves/day, *p* = 0.03). 

Total polyphenol intakes ranged from 47–3187 mg/day across the 10 weeks. Similar to reported vegetable and fruit intakes, the median intakes of total polyphenols were not significantly different from baseline to week 2 (difference −78 (509) mg, *p* = 0.73) or week 10 (−143 (603) mg, *p* = 0.52) of the intervention. The intake of polyphenols from fruit had a non-significant increase from baseline to week 2 (difference +18 (114) mg, *p* = 0.10) and increased significantly from baseline to week 10 (+40 (106) mg, *p* = 0.03). There were no significant differences in intakes of polyphenols from vegetables from baseline to weeks 2 (difference +10 (71) mg, *p* = 0.60) or 10 (+3 (106) mg, *p* = 0.29) (Supplementary Table S2). 

**Figure 1:**
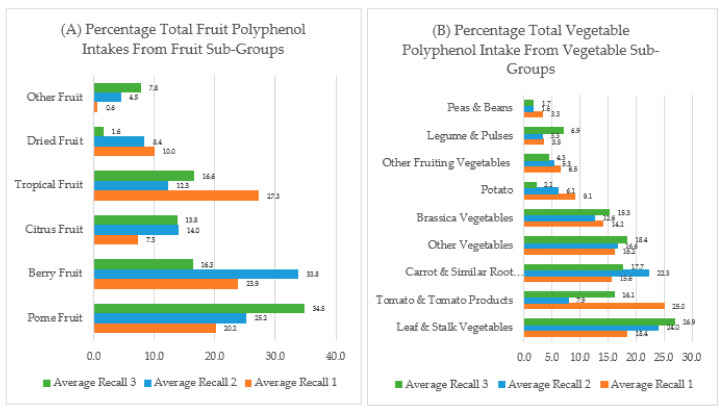
Percentage of total fruit or vegetable polyphenol intakes from sub-groups of fruit and vegetable intakes at baseline, week 2 and week 10. (**A**) Percentage of total fruit polyphenol intake from fruit sub-groups; (**B**) Percentage of total vegetable polyphenol intakes from vegetable sub-groups.

Across each time point average polyphenols from fruits made up 11–20% of total polyphenol intakes and vegetables made up 12–16% of total polyphenol intakes. The majority of fruit polyphenols came from pome fruits (20–35%), while leaf and stalk vegetables contributed to the majority of vegetable polyphenols (18–27%) (Figure 1, Supplementary Table S2). 

### 3.2. Urinary Polyphenol Excretion 

**Figure 2:**
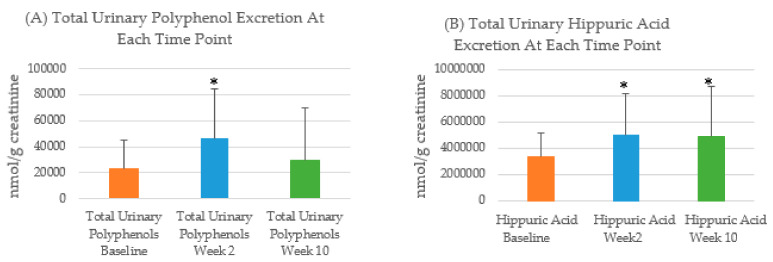
Urinary biomarker excretion at baseline, week 2 and week 10. (**A**) Average (SD) total urinary polyphenol (nmol/g creatinine) excretion at baseline, week 2 and week 10; * significant from baseline; (**B**) Average (SD) hippuric acid excretion (nmol/g creatinine) at baseline, week 2 and week 10; * significant from baseline.

Total urinary polyphenols increased significantly from baseline to week 2 (*p* = 0.004), whereas there was no significant difference in total urinary polyphenols from baseline to week 10 (*p* = 0.52) (Figure 2). Concentrations of urinary hippuric acid increased significantly from baseline to week 2 (*p* = 0.005) and at week 10 (*p* = 0.002). 

### 3.3. Correlations between Dietary Polyphenol Intakes and Urinary Polyphenols 

**Figure 3:**
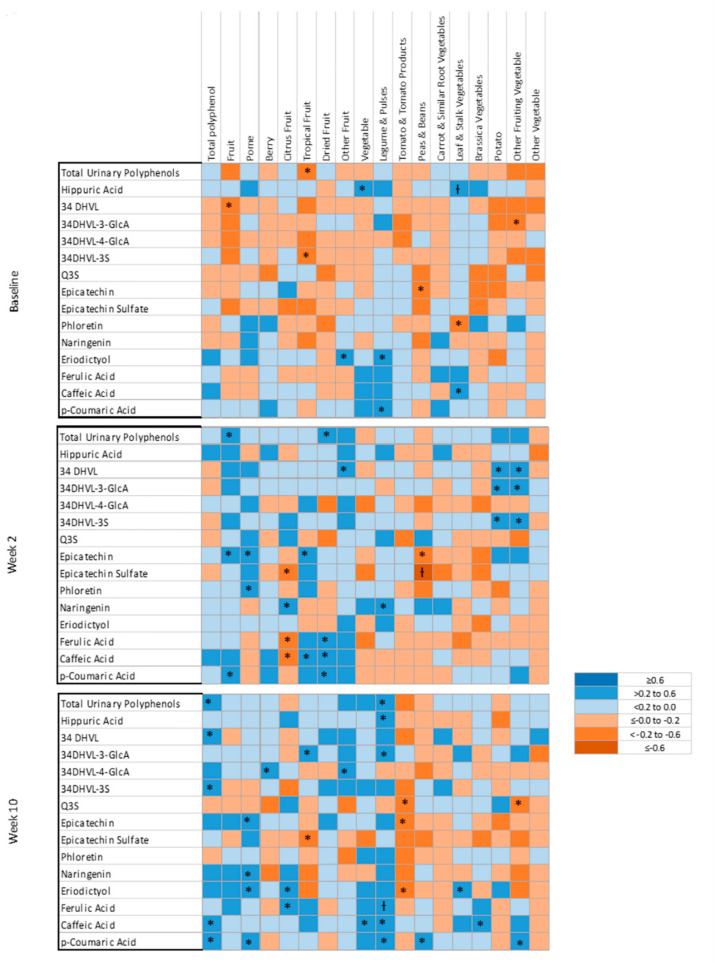
Heat map showing correlations between dietary intake and urinary polyphenol metabolites at baseline, week 2 and week 10. * *p* < 0.05; Ɨ *p* < 0.0028. 34DHVL, 5-(3,4-dihydroxyphenyl)-y-valerolactone; 34DHVL-3-GlcA, 5-(3,4-dihydroxyphenyl)-y-valerolactone 3-O-glucuronide; 34DHVL-4-GlcA, 5-(3,4-dihydroxyphenyl)-y-valerolactone 4-O-glucuronide; 34DHVL-3S, 5-(3,4-dihydroxyphenyl)-y-valerolactone 3-sulphate; Q3S, quercetin-3-O-sulfate.

Correlations between total urinary polyphenols and total polyphenol intakes were only statistically significant at week 10 (*r*_s_ = 0.47, *p* = 0.005), (Figure 3, Supplementary Table S3). Correlations between total urinary polyphenols and vegetable polyphenols were not statistically significant. The correlation between total urinary polyphenols and fruit intake was only significant at week 2 (*r*_s_ = 0.38). Total urinary polyphenols showed statistically significant correlations with polyphenols from tropical fruits (*r*_s_ = −0.34, baseline), dried fruit (*r*_s_ = 0.34, week 2) and legumes and pulses (*r*_s_ = 0.41, week 10).

Urinary hippuric acid showed a moderate, significant correlation with vegetable polyphenols at baseline only (*r*_s_ = 0.39). Statistically significant relationships were also identified between hippuric acid and polyphenols from legumes and pulses (*r*_s_ = 0.36) and leaf and stalk vegetables (*r*_s_ = 0.53), (Figure 3, Supplementary Table S3).

Correlations were further explored to evaluate relationships between dietary polyphenol intakes and individual urinary polyphenol metabolites (Figure 3, Supplementary Table S4). Figure 3 displays the correlations between average dietary total, vegetable and fruit sub-group polyphenols (*n* = 18) and the individual urinary polyphenol metabolites (*n* = 13) explored, of which 60 were significant. Of note, there were nine significant correlations between dietary intake polyphenols and urinary polyphenol metabolites at baseline, 24 at week 2 and 27 at week 10. Consistent correlations were observed between several individual polyphenol metabolites and polyphenol intakes. Epicatechin sulphate and peas and beans polyphenols (*r*_s_ = −0.27 to −0.65) were moderate to strongly, negatively correlated. Eriodictyol was weakly to moderately correlated with total polyphenol intakes (*r*_s_ = 0.10–0.24), pome fruit polyphenols (*r*_s_ = 0.16–0.36) and legume and pulse polyphenols (*r*_s_ = 0.25–0.38). Urinary caffeic acid was consistently correlated with total dietary polyphenol intakes (*r*_s_ = 0.29–0.46).

The sensitivity analysis identified far fewer (*n* = 3) significant correlations. A significant correlation was identified between hippuric acid and leaf and stalk polyphenols at baseline only (*r*_s_ = 0.53), urinary epicatechin sulphate and peas and bean polyphenols (*r*_s_ = −0.65) and lastly at week 10 between urinary ferulic acid and legume and pulse polyphenols (*r*_s_ = 0.55). 

### 3.4. Linear Mixed Model Associations between Urinary Metabolites and Total Polyphenol Intakes

Using linear mixed models, a statistically significant relationship was identified between total urinary polyphenol and total polyphenol intakes (unadjusted model only). This model suggests that every 1mg of total polyphenol intake results in 16.3 nmol/g creatinine excretion of total urinary polyphenols. No other statistically significant relationships were identified (Table 2). 

## 4. Discussion

The current study evaluated relationships between total urinary polyphenols and dietary polyphenol intakes, with a specific focus on polyphenols found in vegetables and fruits. Although significant correlations were identified between total polyphenol intakes, based on self-reported diets, and total urinary polyphenols, individual vegetable and fruit polyphenols and individual polyphenol metabolites as well as vegetable polyphenols and hippuric acid, the strength of the relationships were not consistent across all time points. 

The range in total polyphenol intakes was substantial (47–3187 mg/day), with a median total polyphenol intake of approximately 500 mg/day. A systematic review of cohort and case control studies from Europe, North America, Asia, Australia and South America reported mean total polyphenol intakes in adults as 890 mg/day [36]. Hence, the estimated intakes in the current study are relatively low. However, there has been no estimate of average total polyphenol intakes in Australia, only flavonoid intakes. Dietary polyphenols came from a variety of food sources with more than 60% of total polyphenols coming from food groups other than vegetables and fruits. This is similar to other studies that have identified the main contributors to polyphenol intakes as being tea, coffee, chocolate and red wine [36,37]. 

Reported intakes of citrus fruits, berries, along with leaf/stalk, carrot and similar root vegetable polyphenols increased between baseline and week 2, suggesting increased intakes of vegetables and fruits provided to participants in the first week of the study. Increased intakes of other vegetables and fruits not provided in the vegetable and fruit box such as pome fruits (e.g., apple, pear), other fruit (e.g., grapes, kiwi fruit) and other vegetable (e.g., onion, garlic) polyphenols were also observed. At week 10, increased intakes of citrus fruits, pome fruits, other fruits, legumes and pulses, carrots and similar root vegetables and leaf/stalk vegetables were reported compared to baseline. This suggests that continued consumption of the types of vegetables and fruits provided in the first week vegetable and fruit box occurred, as well as an increased intake of other varieties of vegetables and fruits. Increases in these other varieties of vegetables and fruits is likely due to personal preference, seasonality, availability or affordability of these types of vegetables and fruits in comparison to those provided in the study. Seasonality may have influenced vegetable and fruit choices of participants, as recruitment occurred between August and March (Australian Winter–Autumn), which may have resulted in more or less exposure to some vegetables and fruits depending on the season(s) when they were enrolled in the study. For example, citrus fruit may intake increase in the winter and berry intake in the summer. Although intakes of vegetables and fruits increased, their overall contribution to total polyphenol intakes was minimal (< 20% total polyphenols each), which may explain why there was no significant difference observed in total polyphenol intakes. Additionally, while increasing vegetable and fruit intakes participants may have reduced intakes of other polyphenol rich sources such as alcohol or chocolate intakes.

Significant increases from baseline of total urinary polyphenols (week 2) and hippuric acid (week 2 and 10) were observed even though the change in total polyphenol intakes was not significant. Potentially, the polyphenols in the food consumed at week 2 were more easily absorbed than those consumed at baseline which resulted in a greater excretion of urinary polyphenols [38]. Further research should explore whether there is an ideal time for the collection of spot urinary polyphenols after eating. Hippuric acid excretion has been shown to be lower in people with obesity [39]. This may explain why excretion was significantly higher across the weight loss intervention. Potentially, this may limit hippuric acids applicability as a biomarker in weight loss interventions and could be explored further. 

Inconsistent findings were observed between polyphenol intakes and total urinary polyphenol excretion. However, the correlations between urinary polyphenol concentrations and total polyphenol (week 2 and 10) were similar to those reported in other studies (*r* = 0.15–0.48) [20,40,41]. The dietary assessment method in each of these studies varied from FFQs [20,40], food diaries [40] to 24-h recalls [41]. Of these studies only one used spot urine samples [40], the remaining used 24-h urine samples [20,41]. More consistent relationships between dietary and urinary polyphenols were observed in the studies which used 24-h urine samples [20,41], this expected as 24-h urine samples are likely to capture a large volume and a bigger range of polyphenols compared to spot urine samples due to the varied half-life of urinary polyphenols of 1–24 h [15,21]. However, the collection of 24-h urine samples is not always feasible and practical, which is why understanding more about the application of spot urine samples is required.

Hippuric acid is a naturally occurring metabolite in the urine, which increases with intakes of polyphenol-rich foods [32]. Hippuric acid was the most abundant urinary metabolite and demonstrated a statistically significant correlation with vegetable polyphenols only. Similarly, hippuric acid was identified to be the most abundant metabolite in urine in a previous study that compared urinary metabolite profiles of older (≥50 year old) and younger (≤45 year old) adults in response to both high and low polyphenol diets [42]. The majority of studies that have examined hippuric acid as a potential biomarker have used it as a biomarker of intake of polyphenol-rich food sources, including tea [43,44], cocoa [45], vegetables and fruits [46]. Findings from these studies suggest that because hippuric acid increases with intakes of polyphenol rich food sources that it may be a suitable candidate biomarker for polyphenol intakes. In one study, hippuric acid was explored in 24-h urine samples as a biomarker of vegetables and fruits flavonoid intakes measured using a 3-day weighed food record, with this study reporting significant correlations (*r* = 0.47–0.64, *p* < 0.0001) [47]. Correlations in the current study were weaker than those identified previously by Penczynski et al. [47]. This may be due to the difference in dietary collection methods, a different database being used to assess polyphenol intakes or due to hippuric acid’s varied time needed to reach maximum urinary concentration (1.5–15 h) [48,49] and because fasted spot samples were used, whereas Penczynski et al. [47] used 24-h urine samples. Each of these differences may explain the differences observed between Penczynski et al. and this current study. 

Few consistent correlations were identified between individual urinary polyphenol metabolites and polyphenol intakes in the current study. Consistent, negative correlations were observed between epicatechin sulphate and polyphenols from peas and beans. At the same time, other polyphenol metabolites, such as ferulic acid and citrus fruit polyphenols, had both positive and negative significant correlations. Negative correlations may be influenced by other components of the food matrix which inhibit the absorption or excretion of polyphenols in the urine [18]. The food matrix can also influence the half-life of polyphenols [16] and may explain the varied half-life of polyphenol metabolites (e.g., ferulic acids with a half-life of 2–7 h) [16,50]. This varied half-life may mean that the window to detect a relationship between intakes was missed in some samples and captured in others, resulting in both positive and negative relationships being identified. Additionally, a negative correlation may identify the low specificity of the biomarker for those polyphenols. 

Eriodictyol was consistently, moderately correlated with total polyphenol intakes, pome fruit polyphenols and legume and pulse polyphenols. Similar findings have been observed between eriodictyol and apple intakes (24-h urines) [3], but not for legume and pulse intakes [51]. Urinary caffeic acid was consistently, moderately correlated with total urinary polyphenol intakes. Caffeic acid is naturally present in a variety of polyphenol-rich food sources [52] and has been identified as a potential biomarker for coffee intake [22,53]. The fact that the majority of the total polyphenols came from non-fruit and vegetable sources, such as coffee, may explain why a relationship was observed between total polyphenol intakes and caffeic acid. 

Citrus fruit polyphenols in the current study were correlated with the metabolites naringenin, eriodicytol and ferulic acid. Similar to other studies, naringenin metabolites have also been statistically significant and positively correlated with intakes of citrus fruits measured using 24-h recalls [3,53,54]. Stronger correlations were identified at weeks 2 and 10 when citrus polyphenol intakes were higher. The short half-life of naringenin (1.3–2.9 h) [15] or the long fasting time prior to collection, may mean lower intakes of citrus polyphenols are not reflected in spot urine samples. Phloretin was consistently, moderately correlated with pome fruit polyphenol intakes, except at week 10. Phloretin has been identified as reflecting intakes of pome fruits (apples and pears) previously [3,22,53,54,55]. Therefore, the relationship between phloretin and pome fruit polyphenols was not surprising. Similar findings have been observed using spot urine samples in an older population (35–60 year olds) [22]. All other studies that identified similar relationships between phloretin and apple intakes used 24-h urine samples [3,53,54,55]. Further research is required to determine the reproducibility of these results in spot urine samples. 

The mixed model analyzes identified statistically significant relationships between total urinary polyphenols and polyphenol intakes. These models accounted for the within and between individual differences and by combining samples created a larger sample size which strengthened findings. A relationship between total urinary polyphenols and total polyphenol intakes was identified, however it was only statistically significant in the unadjusted models (where only urinary polyphenols, dietary polyphenol and time were included). Although no confounders influenced the model significantly, the adjustment for age, energy intake, sex and BMI did influence the relationship between urinary polyphenols and polyphenol intake, as adjusted models were no longer significant. The influence of confounders on the adjusted model also follows findings in previous studies which have identified multiple confounders of polyphenol bioavailability and absorption [7,18,19,20]. Although it is not possible to adjust for all confounders, consideration should be given to those that can be accounted for to help further understand the impacts of confounders on findings. 

Limitations of the current study include that a European database (Phenol Explorer) was used to match foods recorded in the 24-h recalls to estimate polyphenol intakes in Australia. There is currently no Australian database that can be used to assess total dietary polyphenol intakes. There is an anthocyanin database [56], however this contains limited foods and is specific to anthocyanins only. The lack of geographic specific databases has been previously identified as limitations [57]. When matching foods from recalls with the Phenol Explorer database it was assumed that some foods contained no polyphenols when there was inadequate information to determine if there were polyphenol containing components and how much of these was included in the reported food item or mixed dish. This is a limitation of the current study, and it may have underestimated total polyphenol intakes. The term total urinary polyphenols refers to only the limited number of urinary polyphenol metabolites that were analyzed in this current study and therefore do not truly reflect the presence of total urinary polyphenol metabolites. Another limitation is that a targeted measurement approach to identify polyphenol metabolites in the urine was used. This approach increases sensitivity to identify metabolites that were actively sought out, however it may mean that other polyphenol metabolites that contributed to total polyphenol intakes may have been missed. This exploratory study was undertaken as part of a weight loss study and therefore was not designed to have power to detect relationships between urinary polyphenol metabolites and polyphenol intakes. Additionally, findings may have been limited as this was a weight loss intervention, and weight loss has been known to alter metabolic processes. This may be reflected in the excretion of hippuric acid, which has previously been identified to be influenced by weight and weight change [39]. Potentially, the grouping of vegetables and fruits into sub-groups may not reflect the variable polyphenol composition present in individual vegetables and fruits, and therefore, this may have also limited the strength of the relationships observed. Lastly, this study focused on polyphenols from vegetables and fruits. As vegetable and fruit polyphenols made up about 40% of total polyphenol intakes of participants in the current study, it is possible that polyphenol intakes from other food groups may explain more of the variation and a greater proportion of the urinary polyphenols than vegetable and fruit polyphenols. Therefore, while total polyphenol intakes were looked at, other polyphenol-rich food sources, such as tea and coffee, were not considered as confounders in statistical models. The primary strength of the current analysis is that it provides a detailed exploration of the relationship between both total and individual polyphenol metabolites and a range of dietary polyphenols in vegetables and fruits and their sub-groups. 

Future studies should explore the application of spot urinary polyphenol analysis to determine its strength, sensitivity and reliability as a biomarker of total polyphenol intake from all foods and beverages, rather than vegetable and fruit polyphenols only. This could be done by conducting studies using repeat spot urine samples collected at multiple times throughout the day to determine the ideal timing for spot sample collection to allow for the most consistent findings. Further research could determine whether the current results can be replicated and could also extend studies to other population groups and cohorts such as Europe and North America, in order to determine the influence of geographical location, the food supply and local dietary culture on polyphenol intakes as well as the reliability and reproducibility of results generally. Total urinary polyphenols appear to be a promising biomarker of total polyphenol intakes and urinary hippuric acid as biomarker of vegetable polyphenols, as long as adjustments are made for intake of other polyphenol-rich food sources, such as tea and coffee. However further research is required. Lastly, if spot urinary polyphenols show consistent and reproducible results, then future studies could trial using spot urinary polyphenols to assess measurement error within calibration studies and interventions. 

## 5. Conclusions

Few consistent findings were observed across time points. However, linear mixed models identified a promising relationship between total urinary polyphenol concentrations and total polyphenol intakes. Overall, spot urine samples may be promising for the identification of relationships between total urinary polyphenols and total polyphenol intakes, hippuric acid and vegetable polyphenol intakes as well as phloretin and pome fruit polyphenols. Further research is required to validate spot urinary polyphenols as biomarkers of polyphenol intakes in a wider variety of polyphenol food sources. 

## Figures and Tables

**Table 1:** Demographic data.

Participant Characteristics (*n* = 34)	Total Participants (*n* = 34)	Males Only (*n* = 16)	Females Only (*n* = 18)
Age (y)	35 (15)	35 (13)	35(16)
Caucasian *n* (%)	22 (65)	13 (81)	9 (50)
Smoking status *n* (%)	1 (3)	1 (6)	0 (0)
BMI (kg/m^2^)	29.0 (2.5)	28.8 (2.4)	29.0 (2.1)

Median (interquartile range); n (%). BMI, Body Mass Index.

**Table 2:** Results of linear mixed model analyzes exploring relationships between total, vegetable and fruit polyphenol intakes and total urinary polyphenols and urinary hippuric acid.

	Total Urinary Polyphenols		Hippuric Acid	
Dietary Intake	Combined Recalls (β)		Combined Recalls (β)	
	Unadjusted	Adjusted	Unadjusted	Adjusted
Total Polyphenols	16.34 (*p* = 0.046) *	15.74 (*p* = 0.07)	644.08 (*p* = 0.35)	634.85 (*p* = 0.38)
Fruit Polyphenols	−12.65 (*p* = 0.72)	−11.96 (*p* = 0.74)	5491.27 (*p* = 0.05)	5170.15 (*p* = 0.07)
Vegetable Polyphenols	124.15 (*p* = 0.10)	127.45 (*p* = 0.10)	6915.57 (*p* = 0.27)	7718.30 (*p* = 0.23)

* *p* < 0.05.

## Supplementary Materials

The following are available online at https://www.mdpi.com/2072-6643/12/11/3431/s1, Table S1: Fruit and vegetable box contents and polyphenol content of box. Table S2: Dietary polyphenol intakes from fruits and vegetables. Table S3: Urinary hippuric acid and total urinary polyphenol biomarkers and dietary intake correlations at baseline, week 2 and week 10. Table S4: Individual urinary metabolites correlation with polyphenol intakes at baseline, week 2 and week 10.
