# Supplementary material for: The Relationship between Dietary Polyphenol Intakes and Urinary Polyphenol Concentrations in Adults Prescribed a High Vegetable and Fruit Diet
Source: Nutrients. 2020 Nov 9;12(11):3431. doi: 10.3390/nu12113431 (PMC7695339; doi:10.3390/nu12113431)
Supplement: Supplementary file 1 [file nutrients-12-03431-s001.pdf]

Supplementary Tables

Supplementary Table S1: Fruit and vegetable box contents and polyphenol content of box.

	Weight (g)	Total Polyphenols (mg)
**Fruit**		
Bananas (x5)	550	14.0
Oranges (x5)	640	313
Frozen Mixed Berries (1 bag)	500	1,090
**Vegetables**		
Carrots (1 bag)	1,000	200
Sweet potato (1 medium)	250	0.8
Half Pumpkin	1,200	20.9
Red capsicum (1 capsicum)	180	1.5
Head of Lettuce	250	20.2
Tomato (1 medium)	110	4.6
Head of Broccoli	300	136
Cucumber (x1)	150	5.9
Zucchini (x1)	210	2.8
Frozen peas and corn (1 bag)	500	0.1
Canned tomatoes (1 tin)	400	17.0

Supplementary Table S2: Dietary Polyphenol Intakes from Fruits and Vegetables

	Baseline (n=34)	***Week 2 (n=34)***	***Week 10 (n=34)***
Total polyphenol intake (mg)	506.0 (543.9)	503.1 (371.1)	517.3 (429.2)
% Fruit polyphenols	2.9 (16.8)	8.5 (29.7)	18.4 (27.7)
Fruit polyphenols (mg)	9.6 (69.9)	64.8 (117.4)	77.9 (110.2)
Pome Fruit Polyphenol(mg)(a)	0.0 (0.9)	0.0 (46.2)	0.0 (84.9)
Berry Fruit Polyphenol (mg)	0.0 (7.8)	0.0 (79.9)	0.0 (0.0)
Citrus Fruit Polyphenols (mg)(b)	0.0 (0.0)	0.0 (5.9)	0.0 (8.9)
Tropical Fruit Polyphenols (mg)(c)	0.7 (2.4)	1.3 (3.4)	1.3 (2.8)
Dried Fruit Polyphenols (mg)	0.0 (0.5)	0.0 (1.1)	0.0 (0.3)
Other Fruit Polyphenols (mg)(d)	0.0 (0.0)	0.0 (0.0)	0.0 (1.0)
% Vegetable polyphenols	8.9 (12.1)	9.8 (12.0)	12.8 (18.9)
Vegetable polyphenols (mg)	39.7 (45.4)	45.2 (48.8)	57.8 (70.5)
Legume & Pulse Polyphenols (mg)(e)	0.0 (0.0)	0.0 (0.001)	0.0 (0.0)
Tomato & Tomato Product Polyphenols (mg)	1.9 (5.0)	1.6 (2.5)	1.7 (3.3)
Peas & Bean Polyphenol (mg)	0.0 (0.01)	0.001 (0.6)	0.0 (0.02)
Carrot & Similar Root Vegetable Polyphenols (mg)(f)	5.4 (13.1)	6.8 (18.3)	4.0 (13.8)
Leaf & Stalk Vegetable Polyphenols (mg)(g)	2.7 (14.7)	3.8 (27.6)	6.4 (18.0)
Brassica Vegetable Polyphenols (mg) (h)	4.0 (13.3)	0.0 (16.6)	6.3 (16.8)
Potato Polyphenols (mg)	0.0 (3.2)	0.0 (0.0)	0.0 (0.0)
Other Fruiting Vegetable Polyphenols (mg) (i)	0.6 (1.2)	1.0 (2.3)	1.0 (3.4)
Other Vegetable Polyphenols (mg) (j)	6.8 (11.7)	0.5 (8.5)	3.1 (12.0)

Median (IQR); NA not applicable

(a) Pome fruits e.g. apples, pears; (b) Citrus fruits e.g. oranges, lemons, limes; (c) Tropical fruits e.g. bananas, pineapple, mangoes; (d) Other fruits e.g. grapes, kiwi fruit; (e) Legume and pulses e.g. chickpeas, black beans, baked beans; (f) Carrot and similar root vegetables e.g. sweet potato, beetroot, ginger; (g) Leaf and stalk vegetables e.g. lettuce, spinach, celery, asparagus; (h) Brassica vegetables e.g. cabbage, broccoli, cauliflower, bok choy; (i) Other fruiting vegetables e.g. capsicum, pumpkin, cucumber, zucchini; (j) Other vegetables e.g. onion, garlic.

Supplementary Table S1: Urinary hippuric acid and total urinary polyphenol biomarkers and dietary intake correlations at baseline, Week 2 and Week 10

	**Total Urinary Hippuric Acid**		
**Dietary intake (mg)**	**Baseline (r_s)**	**Week 2**	**Week 10**
Total polyphenol intake (mg)	0.15 (p=0.40)	0.32 (p=0.06)	0.10 (p=0.58)
Fruit polyphenols	0.03 (p=0.86)	0.28 (p=0.10)	0.14 (p=0.43)
Pome Polyphenols	0.22 (p=0.22)	-0.02 (p=0.93)	0.11 (p=0.53)
Berry Polyphenols	0.15 (p=0.39)	0.32 (p=0.07)	0.13 (p=0.47)
Citrus Fruit Polyphenols	0.19 (p=0.28)	-0.03 (p=0.88)	0.23 (p=0.19)
Tropical Fruit Polyphenols	0.10 (p=0.56)	0.12 (p=0.50)	0.03 (p=0.85)
Dried Fruit Polyphenols	-0.13 (p=0.47)	0.05 (p=0.76)	0.01 (p=0.97)
Other Fruit Polyphenols	0.02 (p=0.89)	0.28 (p=0.10)	0.09 (p=0.60)
Vegetable polyphenols	0.39 (p=0.02)	0.01 (p=0.96)	0.06 (p=0.73)
Legume & Pulses Polyphenols	0.31 (p=0.07)	0.32 (p=0.07)	0.36 (p=0.04)
Tomato & Tomato Products Polyphenols	-0.01 (p=0.97)	-0.03 (p=0.86)	-0.14 (p=0.43)
Peas & Beans Polyphenols	0.11 (p=0.54)	-0.03 (p=0.88)	-0.08 (p=0.64)
Carrot & Similar Root Vegetables Polyphenols	0.19 (p=0.29)	0.25 (p=0.16)	-0.12 (p=0.48)
Leaf & Stalk Vegetables Polyphenols	0.53 (p=0.001)	-0.03 (p=0.87)	-0.03 (p=0.87)
Brassica Vegetables Polyphenols	0.25 (p=0.16)	-0.09 (p=0.60)	0.12 (p=0.49)
Potato Polyphenols	0.16 (p=0.36)	0.02 (p=0.89)	-0.24 (p=0.17)
Other Fruiting Vegetable Polyphenols	0.07 (p=0.69)	0.15 (p=0.40)	0.004 (p=0.98)
Other Vegetable Polyphenols	-0.01 (p=0.95)	-0.26 (p=0.14)	0.07 (p=0.71)
	**Total Urinary Polyphenols**		
	**Baseline (r_s)**	**Week 2**	**Week 10**
Total polyphenol intake (mg)	0.04 (p=0.84)	0.15 (p=0.41)	0.47 (p=0.005)
Fruit polyphenols	-0.28 (p=0.11)	0.38 (p=0.03)	0.02 (p=0.90)
Pome Polyphenols	0.01 (p=0.97)	0.09 (p=0.61)	0.02 (p=0.90)
Berry Polyphenols	-0.06 (p=0.74)	0.14 (p=0.43)	0.08 (p=0.64)
Citrus Fruit Polyphenols	0.09 (p=0.60)	0.04 (p=0.82)	-0.04 (p=0.83)
Tropical Fruit Polyphenols	-0.34 (p=0.046)	0.18 (p=0.30)	0.10 (p=0.57)
Dried Fruit Polyphenols	0.02 (p=0.93)	0.34 (p=0.048)	0.13 (p=0.47)
Other Fruit Polyphenols	-0.04 (p=0.83)	0.32 (p=0.07)	0.29 (p=0.10)
Vegetable polyphenols	-0.10 (p=0.59)	-0.07 (p=0.68)	0.28 (p=0.10)
Legume & Pulses Polyphenols	0.07 (p=0.70)	0.07 (p=0.70)	0.41 (p=0.02)
Tomato & Tomato Products Polyphenols	-0.08 (p=0.65)	0.01 (p=0.96)	-0.24 (p=0.18)
Peas & Beans Polyphenols	-0.12 (p=0.50)	-0.16 (p=0.38)	-0.01 (p=0.96)
Carrot & Similar Root Vegetables Polyphenols	0.06 (p=0.73)	0.02 (p=0.92)	0.14 (p=0.44)
Leaf & Stalk Vegetables Polyphenols	0.14 (p=0.44)	0.01 (p=0.98)	0.03 (p=0.89)
Brassica Vegetables Polyphenols	-0.02 (p=0.92)	0.01 (p=0.95)	0.03 (p=0.87)
Potato Polyphenols	-0.14 (p=0.44)	0.28 (p=0.12)	-0.13 (p=0.46)
Other Fruiting Vegetable Polyphenols	-0.22 (p=0.21)	0.29 (p=0.10)	0.08 (p=0.66)
Other Vegetable Polyphenols	-0.24 (p=0.17)	-0.10 (p=0.56)	0.09 (p=0.63)

Supplementary Table S4: Individual urinary metabolites correlation with polyphenol intakes at baseline, Week 2 and Week 10

	**Baseline**	**Week 2**	**Week 10**
Urine 5-(3,4-dihydroxyphenyl)-y-valerolactone (34 DHVL)			
Total polyphenol intake (mg)	-0.06 (p=0.73)	-0.01 (p=0.96)	0.38 (p=0.03)
Fruit polyphenols	-0.40 (p=0.02)	0.29 (p=0.10)	-0.06 (p=0.74)
Pome Polyphenols	-0.09 (p=0.63)	0.23 (p=0.20)	0.002 (p=0.99)
Berry Polyphenols	-0.11 (p=0.53)	0.08 (p=0.64)	0.08 (p=0.67)
Citrus Fruit Polyphenols	0.08 (p=0.67)	0.14 (p=0.44)	-0.18 (p=0.31)
Tropical Fruit Polyphenols	-0.27 (p=0.12)	0.09 (p=0.63)	0.15 (p=0.40)
Dried Fruit Polyphenols	-0.13 (p=0.45)	0.08 (p=0.64)	0.26 (p=0.14)
Other Fruit Polyphenols	-0.15 (p=0.39)	0.35 (p=0.045)	0.26 (p=0.14)
Vegetable polyphenols	-0.16 (p=0.37)	-0.01 (p=0.95)	0.19 (p=0.29)
Legume & Pulses Polyphenols	0.06 (p=0.74)	0.15 (p=0.40)	0.25 (p=0.15)
Tomato & Tomato Products Polyphenols	-0.15 (p=0.40)	0.16 (p=0.37)	-0.28 (p=0.11)
Peas & Beans Polyphenols	-0.15 (p=0.41)	-0.18 (p=0.31)	-0.004 (p=0.98)
Carrot & Similar Root Vegetables Polyphenols	-0.14 (p=0.44)	0.01 (p=0.95)	0.28 (p=0.10)
Leaf & Stalk Vegetables Polyphenols	0.10 (p=0.56)	0.06 (p=0.72)	-0.09 (p=0.61)
Brassica Vegetables Polyphenols	-0.04 (p=0.80)	-0.15 (p=0.39)	0.07 (p=0.71)
Potato Polyphenols	-0.20 (p=0.26)	0.38 (p=0.03)	-0.16 (p=0.37)
Other Fruiting Vegetable Polyphenols	-0.33 (p=0.06)	0.43 (p=0.01)	0.04 (p=0.84)
Other Vegetable Polyphenols	-0.22 (p=0.20)	-0.16 (p=0.37)	0.27 (p=0.12)
Urine 5-(3,4-dihydroxyphenyl)-y-valerolactone 3-O-glucuronide (34DHVL-3-GlcA)			
Total polyphenol intake (mg)	-0.01 (p=0.97)	-0.11 (p=0.55)	0.08 (p=0.63)
Fruit polyphenols	-0.31 (p=0.07)	0.21 (p=0.24)	0.04 (p=0.81)
Pome Polyphenols	0.08 (p=0.66)	0.19 (p=0.28)	0.07 (p=0.70)
Berry Polyphenols	-0.14 (p=0.45)	0.12 (p=0.48)	0.09 (p=0.60)
Citrus Fruit Polyphenols	-0.002 (p=0.99)	0.10 (p=0.59)	-0.03 (p=0.86)
Tropical Fruit Polyphenols	-0.05 (p=0.76)	0.09 (p=0.63)	0.41 (p=0.02)
Dried Fruit Polyphenols	0.08 (p=0.64)	0.10 (p=0.57)	0.04 (p=0.84)
Other Fruit Polyphenols	0.01 (p=0.95)	0.14 (p=0.42)	0.26 (p=0.14)
Vegetable polyphenols	-0.02 (p=0.91)	0.07 (p=0.67)	0.14 (p=0.43)
Legume & Pulses Polyphenols	0.23 (p=0.19)	-0.17 (p=0.34)	0.39 (p=0.02)
Tomato & Tomato Products Polyphenols	-0.20 (p=0.26)	0.14 (p=0.41)	0.08 (p=0.64)
Peas & Beans Polyphenols	-0.01 (p=0.97)	0.04 (p=0.81)	-0.11 (p=0.52)
Carrot & Similar Root Vegetables Polyphenols	-0.17 (p=0.33)	-0.05 (p=0.76)	-0.09 (p=0.63)
Leaf & Stalk Vegetables Polyphenols	0.14 (p=0.42)	0.13 (p=0.45)	0.22 (p=0.20)
Brassica Vegetables Polyphenols	0.10 (p=0.56)	-0.09 (p=0.63)	-0.17 (p=0.32)
Potato Polyphenols	-0.21 (p=0.24)	0.49 (p=0.003)	0.05 (p=0.76)
Other Fruiting Vegetable Polyphenols	-0.41 (p=0.02)	0.40 (p=0.02)	0.28 (p=0.11)
Other Vegetable Polyphenols	-0.03 (p=0.85)	0.03 (p=0.87)	-0.32 (p=0.07)
Urine 5-(3,4-dihydroxyphenyl)-y-valerolactone 4-O-glucuronide (34DHVL-4-GlcA)			
Total polyphenol intake (mg)	-0.15 (p=0.39)	0.02 (p=0.90)	0.32 (p=0.07)

Fruit polyphenols	-0.24 (p=0.17)	0.15 (p=0.39)	0.19 (p=0.29)
Pome Polyphenols	-0.10 (p=0.56)	0.30 (p=0.08)	0.18 (p=0.31)
Berry Polyphenols	-0.12 (p=0.51)	-0.03 (p=0.85)	0.38 (p=0.03)
Citrus Fruit Polyphenols	-0.07 (p=0.69)	-0.18 (p=0.31)	0.11 (p=0.52)
Tropical Fruit Polyphenols	-0.20 (p=0.26)	0.26 (p=0.14)	-0.19 (p=0.29)
Dried Fruit Polyphenols	-0.12 (p=0.51)	-0.20 (p=0.26)	-0.16 (p=0.37)
Other Fruit Polyphenols	-0.07 (p=0.69)	0.21 (p=0.24)	0.43 (p=0.01)
Vegetable polyphenols	-0.10 (p=0.58)	-0.20 (p=0.27)	0.09 (p=0.63)
Legume & Pulses Polyphenols	-0.09 (p=0.62)	0.11 (p=0.52)	-0.14 (p=0.44)
Tomato & Tomato Products Polyphenols	-0.27 (p=0.13)	-0.17 (p=0.35)	-0.02 (p=0.92)
Peas & Beans Polyphenols	0.19 (p=0.28)	-0.24 (p=0.17)	-0.21 (p=0.23)
Carrot & Similar Root Vegetables Polyphenols	-0.04 (p=0.80)	-0.06 (p=0.74)	-0.10 (p=0.56)
Leaf & Stalk Vegetables Polyphenols	0.13 (p=0.45)	-0.12 (p=0.51)	0.19 (p=0.29)
Brassica Vegetables Polyphenols	0.08 (p=0.64)	-0.21 (p=0.23)	-0.14 (p=0.43)
Potato Polyphenols	-0.11 (p=0.54)	-0.13 (p=0.48)	-0.13 (p=0.48)
Other Fruiting Vegetable Polyphenols	-0.17 (p=0.34)	-0.14 (p=0.42)	-0.02 (p=0.91)
Other Vegetable Polyphenols	0.04 (p=0.84)	0.01 (p=0.94)	-0.16 (p=0.36)
Urine 5-(3,4-dihydroxyphenyl)-y-valerolactone 3-sulphate (34DHVL-3S)			
Total polyphenol intake (mg)	0.02 (p=0.92)	-0.03 (p=0.85)	0.39 (p=0.02)
Fruit polyphenols	-0.29 (p=0.09)	0.30 (p=0.08)	-0.10 (p=0.58)
Pome Polyphenols	0.06 (p=0.76)	0.16 (p=0.38)	-0.06 (p=0.74)
Berry Polyphenols	-0.10 (p=0.57)	0.09 (p=0.60)	0.003 (p=0.99)
Citrus Fruit Polyphenols	0.06 (p=0.75)	0.23 (p=0.19)	-0.22 (p=0.21)
Tropical Fruit Polyphenols	-0.41 (p=0.02)	0.03 (p=0.86)	0.12 (p=0.50)
Dried Fruit Polyphenols	-0.01 (p=0.95)	0.13 (p=0.48)	0.20 (p=0.26)
Other Fruit Polyphenols	-0.07 (p=0.69)	0.32 (p=0.07)	0.22 (p= 0.21)
Vegetable polyphenols	-0.19 (p=0.28)	0.02 (p=0.91)	0.22 (p=0.22)
Legume & Pulses Polyphenols	0.002 (p=0.99)	0.12 (p=0.51)	0.24 (p=0.17)
Tomato & Tomato Products Polyphenols	-0.15 (p=0.39)	0.18 (p=0.32)	-0.25 (p=0.15)
Peas & Beans Polyphenols	-0.12 (p=0.50)	-0.14 (p=0.42)	0.04 (p=0.84)
Carrot & Similar Root Vegetables Polyphenols	-0.02 (p=0.89)	0.01 (p=0.95)	0.28 (p=0.11)
Leaf & Stalk Vegetables Polyphenols	0.03 (p=0.89)	0.08 (p=0.65)	-0.05 (p=0.76)
Brassica Vegetables Polyphenols	0.03 (p=0.88)	0.04 (p=0.83)	0.08 (p=0.65)
Potato Polyphenols	-0.13 (p=0.48)	0.37 (p=0.03)	-0.09 (p=0.62)
Other Fruiting Vegetable Polyphenols	-0.29 (p=0.10)	0.37 (p=0.03)	0.08 (p=0.66)
Other Vegetable Polyphenols	-0.25 (p=0.15)	-0.16 (p=0.38)	0.16 (p=0.38)
Urine quercetin-3-O-sulphate (Q3S)			
Total polyphenol intake (mg)	-0.02 (p=0.93)	-0.04 (p=0.81)	-0.001 (p=0.99)
Fruit polyphenols	-0.18 (p=0.30)	0.07 (p=0.68)	-0.11 (p=0.52)
Pome Polyphenols	-0.18 (p=0.30)	0.25 (p=0.15)	-0.06 (p=0.72)
Berry Polyphenols	-0.21 (p=0.23)	-0.06 (p=0.75)	-0.23 (p=0.20)
Citrus Fruit Polyphenols	0.13 (p=0.47)	0.31 (p=0.08)	0.29 (p=0.09)
Tropical Fruit Polyphenols	0.14 (p=0.43)	-0.08 (p=0.67)	-0.19 (p=0.28)
Dried Fruit Polyphenols	-0.21 (p=0.23)	-0.25 (p=0.15)	0.14 (p=0.44)
Other Fruit Polyphenols	-0.13 (p=0.47)	0.13 (p=0.48)	-0.26 (p=0.13)

Vegetable polyphenols	-0.10 (p=0.56)	0.04 (p=0.83)	0.18 (p=0.31)
Legume & Pulses Polyphenols	0.10 (p=0.58)	0.27 (p=0.12)	-0.03 (p=0.87)
Tomato & Tomato Products Polyphenols	0.16 (p=0.36)	-0.25 (p=0.15)	-0.38 (p=0.03)
Peas & Beans Polyphenols	-0.25 (p=0.16)	0.22 (p=0.21)	0.0003 (p=0.999)
Carrot & Similar Root Vegetables Polyphenols	-0.09 (p=0.62)	0.16 (p=0.38)	0.09 (p=0.62)
Leaf & Stalk Vegetables Polyphenols	0.15 (p=0.41)	-0.15 (p=0.41)	0.11 (p=0.52)
Brassica Vegetables Polyphenols	-0.26 (p=0.14)	-0.07 (p=0.68)	0.06 (p=0.75)
Potato Polyphenols	-0.20 (p=0.27)	-0.14 (p=0.43)	0.27 (p=0.12)
Other Fruiting Vegetable Polyphenols	0.002 (p=0.99)	-0.33 (p=0.06)	-0.35 (p=0.04)
Other Vegetable Polyphenols	-0.29 (p=0.10)	0.05 (p=0.77)	-0.11 (p=0.55)
Urine Epicatechin			
Total polyphenol intake (mg)	-0.05 (p=0.76)	0.07 (p=0.68)	0.28 (p=0.11)
Fruit polyphenols	-0.12 (p=0.48)	0.35 (p=0.04)	0.21 (p=0.23)
Pome Polyphenols	0.01 (p=0.94)	0.45 (p=0.008)	0.36 (p=0.04)
Berry Polyphenols	0.08 (p=0.65)	0.08 (p=0.65)	0.04 (p=0.83)
Citrus Fruit Polyphenols	0.34 (p=0.05)	-0.06 (p=0.75)	0.12 (p=0.49)
Tropical Fruit Polyphenols	-0.05 (p=0.77)	0.42 (p=0.01)	-0.01 (p=0.97)
Dried Fruit Polyphenols	-0.15 (p=0.39)	0.02 (p=0.89)	0.22 (p=0.20)
Other Fruit Polyphenols	0.05 (p=0.79)	0.15 (p=0.41)	0.15 (p=0.39)
Vegetable polyphenols	-0.05 (p=0.77)	-0.06 (p=0.72)	0.19 (p=0.27)
Legume & Pulses Polyphenols	0.15 (p=0.40)	0.03 (p=0.89)	0.31 (p=0.08)
Tomato & Tomato Products Polyphenols	0.04 (p=0.83)	0.04 (p=0.84)	-0.39 (p=0.02)
Peas & Beans Polyphenols	-0.41 (p=0.02)	-0.39 (p=0.02)	-0.12 (p=0.49)
Carrot & Similar Root Vegetables Polyphenols	-0.08 (p=0.65)	-0.07 (p=0.69)	-0.005 (p=0.98)
Leaf & Stalk Vegetables Polyphenols	0.17 (p=0.33)	-0.16 (p=0.36)	0.11 (p=0.55)
Brassica Vegetables Polyphenols	-0.23 (p=0.19)	-0.21 (p=0.23)	-0.05 (p=0.80)
Potato Polyphenols	-0.34 (p=0.05)	0.20 (p=0.26)	-0.21 (p=0.23)
Other Fruiting Vegetable Polyphenols	-0.003 (p=0.99)	0.24 (p=0.17)	-0.07 (p=0.68)
Other Vegetable Polyphenols	-0.10 (p=0.58)	0.08 (p=0.67)	-0.003 (p=0.99)
Urine Epicatechin Sulphate			
Total polyphenol intake (mg)	0.18 (p=0.32)	-0.06 (p=0.73)	0.06 (p=0.75)
Fruit polyphenols	-0.21 (p=0.23)	0.08 (p=0.64)	-0.001 (p=0.995)
Pome Polyphenols	-0.17 (p=0.32)	0.25 (p=0.16)	0.29 (p=0.09)
Berry Polyphenols	-0.02 (p=0.90)	-0.07 (p=0.69)	-0.19 (p=0.28)
Citrus Fruit Polyphenols	-0.20 (p=0.25)	-0.37 (p=0.03)	-0.03 (p=0.87)
Tropical Fruit Polyphenols	-0.25 (p=0.15)	0.33 (p=0.06)	-0.38 (p=0.03)
Dried Fruit Polyphenols	-0.16 (p=0.36)	0.19 (p=0.29)	0.06 (p=0.74)
Other Fruit Polyphenols	-0.02 (p=0.91)	0.14 (p=0.44)	-0.14 (p=0.43)
Vegetable polyphenols	0.02 (p=0.89)	-0.26 (p=0.14)	-0.20 (p=0.26)
Legume & Pulses Polyphenols	0.02 (p=0.89)	0.07 (p=0.68)	0.02 (p=0.90)
Tomato & Tomato Products Polyphenols	0.05 (p=0.76)	0.06 (p=0.75)	-0.28 (p=0.10)
Peas & Beans Polyphenols	-0.29 (p=0.09)	-0.65 (p<0.001)	-0.27 (p=0.12)
Carrot & Similar Root Vegetables Polyphenols	0.10 (p=0.56)	-0.33 (p=0.06)	-0.18 (p=0.32)

Leaf & Stalk Vegetables Polyphenols	0.13 (p=0.45)	-0.08 (p=0.66)	-0.10 (p=0.59)
Brassica Vegetables Polyphenols	-0.26 (p=0.13)	-0.32 (p=0.07)	-0.21 (p=0.24)
Potato Polyphenols	-0.05 (p=0.77)	0.13 (p=0.47)	-0.15 (p=0.39)
Other Fruiting Vegetable Polyphenols	0.09 (p=0.62)	0.07 (p=0.68)	-0.21 (p=0.22)
Other Vegetable Polyphenols	-0.05 (p=0.77)	0.14 (p=0.43)	-0.13 (p=0.46)
Urine Phloretin			
Total polyphenol intake (mg)	-0.14 (p=0.44)	0.04 (p=0.82)	-0.02 (p=0.91)
Fruit polyphenols	0.03 (p=0.85)	0.14 (p=0.44)	0.15 (p=0.41)
Pome Polyphenols	0.31 (p=0.07)	0.36 (p=0.04)	0.09 (p=0.60)
Berry Polyphenols	0.20 (p=0.26)	0.11 (p=0.54)	-0.17 (p=0.34)
Citrus Fruit Polyphenols	-0.13 (p=0.47)	-0.17 (p=0.35)	0.09 (p=0.63)
Tropical Fruit Polyphenols	-0.12 (p=0.49)	0.23 (p=0.19)	-0.002 (p=0.99)
Dried Fruit Polyphenols	-0.21 (p=0.23)	-0.16 (p=0.38)	0.03 (p=0.86)
Other Fruit Polyphenols	0.15 (p=0.39)	0.15 (p=0.40)	-0.20 (p=0.27)
Vegetable polyphenols	0.15 (p=0.40)	0.16 (p=0.37)	0.22 (p=0.21)
Legume & Pulses Polyphenols	0.13 (p=0.47)	0.10 (p=0.57)	0.33 (p=0.06)
Tomato & Tomato Products Polyphenols	-0.11 (p=0.55)	-0.16 (p=0.38)	-0.23 (p=0.19)
Peas & Beans Polyphenols	-0.09 (p=0.61)	-0.28 (p=0.11)	-0.01 (p=0.96)
Carrot & Similar Root Vegetables Polyphenols	0.18 (p=0.31)	0.14 (p=0.43)	-0.14 (p=0.43)
Leaf & Stalk Vegetables Polyphenols	-0.37 (p=0.03)	0.07 (p=0.68)	0.17 (p=0.33)
Brassica Vegetables Polyphenols	0.27 (p=0.12)	-0.11 (p=0.53)	0.05 (p=0.80)
Potato Polyphenols	0.06 (p=0.74)	-0.21 (p=0.24)	0.13 (p=0.47)
Other Fruiting Vegetable Polyphenols	0.24 (p=0.18)	0.05 (p=0.78)	-0.14 (p=0.43)
Other Vegetable Polyphenols	0.02 (p=0.92)	-0.11 (p=0.53)	0.02 (p=0.93)
Urine Gallic Acid (not applicable as only 1 participant excreted gallic acid)			
Urine Naringenin			
Total polyphenol intake (mg)	-0.05 (p=0.78)	0.12 (p=0.50)	0.25 (p=0.15)
Fruit polyphenols	-0.11 (p=0.55)	0.09 (p=0.61)	0.21 (p=0.22)
Pome Polyphenols	0.30 (p=0.08)	-0.13 (p=0.48)	0.39 (p=0.02)
Berry Polyphenols	0.13 (p=0.47)	0.16 (p=0.38)	-0.22 (p=0.22)
Citrus Fruit Polyphenols	-0.08 (p=0.66)	0.40 (p=0.02)	0.23 (p=0.18)
Tropical Fruit Polyphenols	-0.26 (p=0.14)	-0.14 (p=0.43)	-0.28 (p=0.11)
Dried Fruit Polyphenols	-0.05 (p=0.76)	-0.13 (p=0.45)	0.18 (p=0.30)
Other Fruit Polyphenols	0.02 (p=0.92)	0.13 (p=0.46)	-0.15 (p=0.41)
Vegetable polyphenols	-0.11 (p=0.52)	0.22 (p=0.22)	-0.03 (p=0.88)
Legume & Pulses Polyphenols	0.04 (p=0.84)	0.41 (p=0.02)	0.23 (p=0.18)
Tomato & Tomato Products Polyphenols	0.04 (p=0.83)	0.11 (p=0.52)	-0.24 (p= 0.18)
Peas & Beans Polyphenols	-0.32 (p=0.07)	0.20 (p=0.25)	0.16 (p=0.36)
Carrot & Similar Root Vegetables Polyphenols	0.26 (p=0.14)	0.33 (p=0.06)	-0.02 (p=0.93)
Leaf & Stalk Vegetables Polyphenols	-0.15 (p=0.39)	-0.002 (p=0.99)	-0.07 (p=0.71)
Brassica Vegetables Polyphenols	-0.14 (p=0.44)	0.06 (p=0.76)	0.03 (p=0.85)
Potato Polyphenols	-0.06 (p=0.74)	-0.01 (p=0.96)	-0.12 (p=0.49)

Other Fruiting Vegetable Polyphenols	0.09 (p=0.60)	0.03 (p=0.88)	-0.21 (p=0.22)
Other Vegetable Polyphenols	-0.08 (p=0.64)	-0.08 (p=0.66)	-0.002 (p=0.99)
Urine Eriodictyol			
Total polyphenol intake (mg)	0.24 (p=0.17)	0.10 (p=0.58)	0.23 (p=0.20)
Fruit polyphenols	0.11 (p=0.52)	0.12 (p=0.51)	0.30 (p=0.08)
Pome Polyphenols	0.26 (p=0.14)	0.16 (p=0.37)	0.36 (p=0.04)
Berry Polyphenols	0.12 (p=0.48)	0.14 (p=0.42)	0.16 (p=0.38)
Citrus Fruit Polyphenols	0.19 (p=0.29)	0.14 (p=0.42)	0.36 (p=0.04)
Tropical Fruit Polyphenols	0.09 (p=0.62)	-0.14 (p=0.44)	-0.21 (p=0.22)
Dried Fruit Polyphenols	-0.001 (p=0.996)	-0.10 (p=0.56)	0.09 (p=0.61)
Other Fruit Polyphenols	0.39 (p=0.02)	0.21 (p=0.24)	0.19 (p=0.28)
Vegetable polyphenols	0.02 (p=0.93)	-0.15 (p=0.41)	0.29 (p=0.10)
Legume & Pulses Polyphenols	0.38 (p=0.03)	0.28 (p=0.11)	0.25 (p=0.15)
Tomato & Tomato Products Polyphenols	0.04 (p=0.83)	-0.03 (p=0.89)	-0.36 (p=0.04)
Peas & Beans Polyphenols	-0.11 (p=0.55)	0.04 (p=0.84)	-0.07 (p=0.69)
Carrot & Similar Root Vegetables Polyphenols	0.06 (p=0.72)	0.04 (p=0.81)	-0.06 (p=0.76)
Leaf & Stalk Vegetables Polyphenols	0.19 (p=0.29)	-0.19 (p=0.28)	0.36 (p=0.04)
Brassica Vegetables Polyphenols	-0.04 (p=0.84)	-0.31 (p=0.07)	-0.12 (p=0.49)
Potato Polyphenols	-0.34 (p=0.05)	0.09 (p=0.62)	0.21 (p=0.23)
Other Fruiting Vegetable Polyphenols	0.12 (p=0.49)	-0.06 (p=0.75)	-0.33 (p=0.06)
Other Vegetable Polyphenols	-0.07 (p=0.70)	-0.04 (p=0.84)	-0.14 (p=0.42)
Urine Ferulic Acid			
Total polyphenol intake (mg)	0.18 (p=0.30)	0.13 (p=0.48)	0.14 (p=0.42)
Fruit polyphenols	-0.11 (p=0.55)	0.18 (p=0.31)	0.28 (p=0.11)
Pome Polyphenols	-0.001 (p=0.997)	-0.17 (p=0.34)	0.18 (p=0.30)
Berry Polyphenols	0.07 (p=0.69)	0.18 (p=0.32)	-0.06 (p=0.73)
Citrus Fruit Polyphenols	-0.04 (p=0.83)	-0.37 (p=0.03)	0.35 (p=0.04)
Tropical Fruit Polyphenols	-0.12 (p=0.49)	0.33 (p=0.05)	0.28 (p=0.11)
Dried Fruit Polyphenols	-0.11 (p=0.52)	0.37 (p=0.03)	-0.08 (p=0.66)
Other Fruit Polyphenols	-0.002 (p=0.99)	0.20 (p= 0.26)	0.11 (p=0.55)
Vegetable polyphenols	0.24 (p=0.18)	-0.23 (p=0.19)	0.25 (p=0.16)
Legume & Pulses Polyphenols	0.22 (p=0.21)	0.02 (p=0.93)	0.55 (p=0.001)
Tomato & Tomato Products Polyphenols	0.11 (p=0.52)	-0.14 (p=0.42)	-0.04 (p=0.81)
Peas & Beans Polyphenols	-0.16 (p=0.36)	-0.10 (p=0.57)	0.06 (p=0.73)
Carrot & Similar Root Vegetables Polyphenols	0.28 (p=0.11)	-0.06 (p=0.75)	-0.12 (p=0.50)
Leaf & Stalk Vegetables Polyphenols	0.20 (p=0.26)	-0.20 (p=0.25)	0.03 (p=0.87)
Brassica Vegetables Polyphenols	-0.02 (p=0.92)	-0.02 (p=0.91)	0.25 (p=0.15)
Potato Polyphenols	0.06 (p=0.75)	-0.11 (p=0.54)	-0.16 (p=0.37)
Other Fruiting Vegetable Polyphenols	0.07 (p=0.71)	-0.06 (p=0.74)	0.12 (p=0.52)
Other Vegetable Polyphenols	-0.01 (p=0.94)	-0.04 (p=0.84)	-0.11 (p=0.53)
Urine Caffeic Acid			
Total polyphenol intake (mg)	0.29 (p=0.09)	0.29 (p=0.10)	0.46 (p=0.01)

Fruit polyphenols	-0.04 (p=0.81)	0.22 (p=0.21)	0.19 (p=0.28)
Pome Polyphenols	-0.18 (p=0.31)	-0.10 (p=0.58)	0.02 (p=0.89)
Berry Polyphenols	0.04 (p=0.80)	0.25 (p=0.16)	0.19 (p=0.29)
Citrus Fruit Polyphenols	0.12 (p=0.49)	-0.44 (p=0.01)	0.14 (p=0.44)
Tropical Fruit Polyphenols	0.11 (p=0.52)	0.36 (p=0.04)	0.27 (p=0.12)
Dried Fruit Polyphenols	-0.15 (p=0.41)	0.35 (p=0.045)	0.10 (p=0.58)
Other Fruit Polyphenols	-0.03 (p=0.88)	0.23 (p=0.18)	0.10 (=0.57)
Vegetable polyphenols	0.24 (p=0.18)	-0.09 (p=0.60)	0.43 (p=0.01)
Legume & Pulses Polyphenols	0.33 (p=0.06)	-0.11 (p=0.52)	0.43 (p=0.01)
Tomato & Tomato Products Polyphenols	0.09 (p=0.62)	-0.19 (p=0.28)	0.02 (p=0.89)
Peas & Beans Polyphenols	0.01 (p=0.97)	-0.15 (p=0.40)	0.05 (p=0.78)
Carrot & Similar Root Vegetables Polyphenols	-0.01 (p=0.95)	0.04 (p=0.84)	-0.11 (p=0.54)
Leaf & Stalk Vegetables Polyphenols	0.46 (p=0.01)	-0.08 (p=0.65)	0.21 (p=0.22)
Brassica Vegetables Polyphenols	0.02 (p=0.91)	-0.03 (p=0.85)	0.39 (p=0.02)
Potato Polyphenols	-0.01 (p=0.95)	0.06 (p=0.75)	-0.19 (p=0.28)
Other Fruiting Vegetable Polyphenols	-0.001 (p=0.997)	0.09 (p=0.59)	0.28 (p=0.10)
Other Vegetable Polyphenols	0.13 (p=0.46)	-0.05 (p=0.80)	-0.02 (p=0.90)
Urine p-Coumaric Acid			
Total polyphenol intake (mg)	0.16 (p=0.36)	0.18 (p=0.32)	0.38 (p=0.03)
Fruit polyphenols	0.07 (p=0.71)	0.40 (p=0.02)	0.19 (p=0.28)
Pome Polyphenols	0.08 (p=0.66)	-0.06 (p=0.75)	0.43 (p=0.01)
Berry Polyphenols	0.20 (p=0.25)	0.34 (p=0.05)	-0.04 (p=0.81)
Citrus Fruit Polyphenols	0.18 (p=0.31)	-0.18 (p=0.32)	0.12 (p=0.49)
Tropical Fruit Polyphenols	-0.01 (p=0.94)	0.31 (p=0.07)	0.01 (p=0.97)
Dried Fruit Polyphenols	0.08 (p=0.64)	0.39 (p=0.02)	-0.01 (p=0.94)
Other Fruit Polyphenols	0.07 (p=0.70)	0.23 (p=0.19)	0.09 (p=0.60)
Vegetable polyphenols	0.29 (p=0.10)	-0.10 (p=0.56)	0.31 (p=0.08)
Legume & Pulses Polyphenols	0.35 (p=0.04)	-0.15 (p=0.40)	0.42 (p=0.01)
Tomato & Tomato Products Polyphenols	0.18 (p=0.30)	-0.14 (p=0.43)	-0.05 (p=0.79)
Peas & Beans Polyphenols	-0.13 (p=0.46)	-0.14 (p=0.42)	0.35 (p=0.04)
Carrot & Similar Root Vegetables Polyphenols	0.21 (p=0.23)	-0.09 (p=0.62)	0.03 (p=0.88)
Leaf & Stalk Vegetables Polyphenols	0.13 (p=0.46)	0.002 (p=0.99)	0.07 (p=70)
Brassica Vegetables Polyphenols	0.0002 (p=0.999)	0.13 (p=0.46)	0.17 (p=0.34)
Potato Polyphenols	-0.07 (p=0.69)	0.14 (p=0.44)	-0.08 (p=0.65)
Other Fruiting Vegetable Polyphenols	0.16 (p=0.37)	0.26 (p=0.14)	0.38 (p=0.03)
Other Vegetable Polyphenols	-0.11 (p=0.54)	-0.16 (p=0.38)	-0.13 (p=0.46)
